# Alport Syndrome–Associated Pathogenic *COL4A4* Variant in Sisters With Chronic Kidney Disease: Clinical Findings and Integrative Network Analysis

**DOI:** 10.1155/ijog/8868521

**Published:** 2026-05-04

**Authors:** Bakhtawar Farooq, Zahid Habib Qureshi, Muhammad Tahir Khan, Muhammad Faisal, Madeeha Shahzad Lodhi

**Affiliations:** ^1^ Institute of Molecular Biology and Biotechnology, The University of Lahore, Lahore, Pakistan, uol.edu.pk; ^2^ Biochemistry Department, Nishtar Medical University, Multan, Pakistan, nmu.edu.pk; ^3^ Physiology Department, Multan Medical and Dental College, Multan, Pakistan; ^4^ Institute of Plant Breeding and Biotechnology, MNS University of Agriculture, Multan, Pakistan, mnsuam.edu.pk

**Keywords:** Alport syndrome, CKD, GBM, protein–protein interactions

## Abstract

**Background:**

Pathogenic variants in the *COL4A4* gene lead to Alport syndrome, a hereditary kidney disorder characterized by deficiencies in the glomerular basement membrane (GBM), progressive renal failure, and associated visual and auditory dysfunctions.

**Objective:**

This study is aimed at detecting genetic variants and conducting an integrative network analysis to elucidate their involvement in Alport syndrome and chronic kidney disease (CKD).

**Methodology:**

The enrolled patients were biological sisters. Clinical and pathological data were collected using laboratory tests. Genetic testing was performed to identify the variants via whole‐exome sequencing (WES). The pathogenicity of the detected variant was confirmed using different computational approaches. The detected variants were classified according to the ACMG criteria. The interaction networks of CKD, Alport syndrome, and *COL4A4* were examined using STRING v11.5, whereas pathways were analyzed by KEGG and STRING pathways analysis.

**Results:**

Patients were diagnosed with CKD of Stage 5. The ultrasound results were not clear due to kidney fibrosis. WES revealed an 18‐base pair deletion in the *COL4A4* gene (chr2: g.227958874_227958891del). The detected variant was classified as pathogenic by ACMG criteria. Online Mendelian Inheritance in Man (OMIM) reveals that the variant in *COL4A4* is due to autosomal recessive Alport syndrome 2. Sensorineural hearing loss was observed in two family members of the patients. These results show the existence of Alport syndrome in their family. The disease–protein interaction by STRING reveals that the COL4A4 protein is strongly associated with both CKD and Alport syndrome diseases. KEGG analysis indicates a consistent association of PI3K‐Akt and AGE‐RAGE pathways among both diseases.

**Conclusion:**

Dysfunction of *COL4A4* leads to disruptions in GBM structure and signaling, which hasten CKD in Alport syndrome. Genetic testing is essential for identifying the exact cause of the disease, which aids in early diagnosis and control to spread within families.

## 1. Introduction

Alport syndrome is the second most common cause of chronic kidney disease (CKD), followed by polycystic kidney disease [1]. The glomerular basement membrane (GBM) and the basement membranes in other organs, such as the eye and ear, show structural abnormalities and dysfunctions in response to Alport syndrome. Patients with Alport syndrome commonly experience progressive renal function loss accompanied by sensorineural hearing impairment, various visual defects, and distinctive ultrastructural changes in the GBM [2].

The selectively permeable glomerular filtration barrier of GBM is a key component of the extracellular matrix (ECM) and acts as an essential barrier that prevents the passage of blood cells and proteins from the bloodstream into the urine. This sheet‐like ECM primarily consists of four major macromolecules: laminin, Type IV collagen, nidogen, and heparan sulfate proteoglycan (agrin). Type IV collagen, which constitutes about half of the total protein mass in GBM, is vital for maintaining the integrity of the basement membrane. Three different heterotrimers, *α*1*α*1*α*2, *α*3*α*4*α*5, and *α*5*α*5*α*6.3, are formed by the assembly of six genetically diverse *α*1–*α*6 Type IV collagen chains [3].

Alport syndrome is caused by mutations in *COL4A3*, *COL4A4*, and *COL4A5* genes [1]. Pathogenic variants in the *COL4A4* gene result in defective Type IV collagen chains *α*4. These structural defects lead to a dysfunctional GBM, resulting in both proteinuria and hematuria [4]. The pathophysiological consequences include chronic inflammation followed by fibrosis. The association between Type IV collagen abnormalities and renal inflammation and fibrosis is increasing the relevance of targeted anti‐inflammatory therapies in Alport syndrome and progressive forms of CKD [5].

Hematuria, increased renal failure, hearing impairment, and eye abnormalities characterize Alport syndrome, a hereditary kidney disorder. Estimates of Alport syndrome prevalence vary: Historical reports suggest approximately 1 in 5000–50,000 individuals, whereas recent genomic studies estimate pathogenic *COL4A5* variants in approximately 1 in 2300 individuals; heterozygous *COL4A3*/*COL4A4* variants are more frequent, reflecting the broader Alport spectrum [4]. The 15% of Alport syndrome cases are autosomal recessive, resulting from a combination of two polymorphisms in either *COL4A3* or *COL4A4* on different chromosomes, whereas the majority, 85%, is X‐linked, caused by pathogenic variants in *COL4A4* [6]. By the time they reach their twenties, most men and women with recessive Alport syndrome have experienced renal failure.

Individuals with a heterozygous variation in *COL4A4* are carriers of recessive Alport syndrome, which is also referred to as autosomal dominant Alport syndrome or thin basement membrane nephropathy (TBMN) [2]. Although as much as 10% of these heterozygotes may experience renal failure by the age of 60, they typically maintain normal levels of blood pressure, kidney function, hematuria, and proteinuria. Additionally, they do not exhibit any issues with vision or hearing loss. The presence of thinned glomerular membranes in biopsies from normal transplant donor kidneys suggests that roughly 1% of the population carries a heterozygous *COL4A4* variant, which aligns with the incidence of autosomal recessive Alport syndrome [7].

Alport syndrome is diagnosed when hematuria, renal failure, or hearing loss persist, especially in individuals with a family history of these conditions. Alport syndrome is specifically associated with lenticonus, fleck retinopathy, and kidney biopsy findings showing a lamellated or uniformly thinner GBM [8]. Screening for hematuria can detect heterozygotes for *COL4A3* and *COL4A4*; however, many individuals with pathogenic variants in *COL4A3–COL4A5* remain undiagnosed [4]. Genetic testing is essential for identifying the exact cause of disease, especially when laboratory testing fails to diagnose disease. Therefore, this study is aimed at detecting the real cause of disease by genetic testing and integrative network analysis to elucidate their involvement in Alport syndrome and CKD.

## 2. Materials and Methods

### 2.1. Clinical Background of the Patient

Two affected sisters were recruited in a local hospital. Ethical approvals were obtained from the Institute of Molecular Biology and Biotechnology, The University of Lahore (CRiMM/23/Research/39), Nishtar Medical University (IRB No. 13323/NMU), and Ibn‐e‐Siena Research Institute (C‐76‐1028‐01), Multan, Pakistan. Written informed consent was obtained from participants. The demographic profile and relevant medical history were collected using a specifically designed proforma. The clinical and pathological data were collected using laboratory tests, such as creatinine, estimated glomerular filtration rates (eGFR), and electrolytes. Key clinical features are summarized in Table 1. Furthermore, CKD was confirmed by the CKD‐EPI equation. The equation of the CKD‐EPI equation is given below.

eGFR=142×minstandardized Scr/K,1 α∗maxstandardized Scr/K,1−1.2000.99381.012∗ age in years∗ if female

where Scr is the serum creatinine in mg/dL; K is equal to 0.7 (females) or 0.9 (males); *α* is equal to −0.241 (females) or −0.302 (males); min indicates the minimum of Scr/k or 1; and max indicates the maximum of Scr/k or 1.

**Table 1:** Clinical history of CKD patients.

Patient (P)	Gender (M/F)	Age (years)	Creatinine (mg/dL)	eGFR (mL/min/1.73 m^2^)	Serum electrolyte (mmol/L)	Clinical history
P1	F	20	6.9 mg/dL	8 mL/min/1.73 m^2^	Bicarbonate: (20 mmol/L) Na+:(130 mmol/L) K+: (4.2 mmol/L)	Kidney disorder, hypertension, underwent kidney transplant
P2	F	18	9.5 mg/dL	6 mL/min/1.73 m^2^	Bicarbonate:(17.8 mmol/L) Na+:(128 mmol/L) K+: (5.3 mmol/L)	Mesangial glomerulonephritis, hypertension, anemia, kidney disorder

### 2.2. Whole‐Exome Sequencing and Verification of Variants

Exome sequencing was conducted at Dante Labs Genomics in Dubai Silicon Oasis, Building B3, United Arab Emirates. The purpose of the exome sequencing was to verify variants present in the exome (protein‐coding) region, which is thought to encompass a considerable number of disease‐associated variants. Genomic DNA was extracted, fragmented, and subjected to targeted gene capture of the human exome using a custom capture kit. The resulting indexed libraries were sequenced to a depth of 100× using 2 × 150 chemistry on the Illumina sequencing platform. Primary and secondary analyses were executed on the Illumina DRAGEN platform. The sequences generated were aligned with the human reference genome (GRCh37/hg19) utilizing DRAGEN. For tertiary analysis, various disease databases, including ClinVar, OMIM, GWAS, HGMD, and SwissVar, were employed [9]. Common variants were filtered based on their allele frequency in the 1000 Genomes Project Phase 3, ExAC, EVS, and dbSNP147.

### 2.3. Data Analysis and Annotation of WES

After sequencing, the raw data from the NovaSeq X were directly uploaded to Illumina′s online platform (BaseSpace Sequence Hub) for genomic data analysis. The bcl2fastq2 conversion software was used to generate FASTQ files. The secondary bioinformatics analysis was performed on Illumina′s online platform, known as DRAGEN Bio‐IT. The reads obtained during sequencing were utilized in the alignment process to create SAM/BAM files [10]. The identified variant was confirmed via Sanger sequencing. The detected variants were classified according to the ACMG criteria by a website GeneBe (https://genebe.net/).

### 2.4. In Silico Pathogenicity Prediction for Gene Mutations

The impact of identified mutation on the protein structure and function was also analyzed by Mutation Taster (https://www.mutationtaster.org/), SIFT(https://sift.bii.a-star.edu.sg/), PolyPhen2 (http://genetics.bwh.harvard.edu/pph2/), FATHMM (https://fathmm.biocompute.org.uk/), I‐Mutant2 (https://folding.biofold.org/i-mutant/i-mutant2.0.html), and AlphaFold DB (https://alphafold.ebi.ac.uk/) [11].

### 2.5. Protein–Protein Interaction (PPI) Network Analysis

PPI and disease–protein interaction network analysis were performed using the online gene/protein interaction retrieval website STRING (http://www.string-db.org/), and visualization of the interaction network was accomplished using Cytoscape software (https://cytoscape.org/) [12, 13].

### 2.6. Pathway Mapping and Enrichment Analysis

To explore the biological functions of pathogenic genes, KEGG (Kyoto Encyclopedia of Genes and Genomes) pathways [14] were utilized by their UniProt IDs. This analysis helped to elucidate how pathogenic variants may influence disease‐related processes and highlighted common pathways among the proteins analyzed.

## 3. Results

### 3.1. Clinical History and Evaluation

The enrolled patients (P1 and P2) were biological sisters, born as a result of a consanguineous marriage and diagnosed with CKD in a local hospital. The family of patients was reported to have multiple health issues: Her mother and uncle (father′s brother) have CKD and diabetes, her father has a heart problem, her sister has GBA deficiency with bone symptoms, and a niece and a nephew (siblings), also born from a consanguineous marriage, are exhibiting chromosomal abnormalities and diagnosed with sensorineural deafness. The grandparents and other family members remained unaffected.

In addition, the clinical tests including elevated serum creatinine levels and low eGFR for more than 3 months, confirm Stage 5 CKD in a 20‐year‐old P1 patient and an 18‐year‐old P2 patient (Table 1). Clinical history revealed that both patients (P1 and P2) were suffering from hypertension and kidney disorder, whereas the P2 patient was also suffering from mesangial glomerulonephritis and anemia. The results of the ultrasound show that the kidneys were atrophied (shrunken) and brighter (fibrosis), which makes it difficult to determine the original cause of the kidney failure. Further, patients were not showing any signs of hearing loss and specific eye abnormalities. Therefore, genetic testing was performed to observe the actual cause of CKD. A 6‐month follow‐up reveals that Patient P1 developed severe kidney damage, initially had dialysis, but underwent kidney transplant due to kidney failure, and the patient P2 died (6 months after genetic testing) due to severe kidney damage. The clinical investigation supported the hypothesis that the family has genetic disorders.

### 3.2. WES Confirms 18 Bp Deletion in *COL4A4* and Alport Syndrome

The whole‐exome sequencing was conducted to identify the genetic mutations. WES revealed that both patients were shown to have a homozygous 18‐base pair deletion in the 20th exon of *COL4A4* gene (chr2: g.227958874_227958891del) as shown in Table 2. This deletion results in the in‐frame deletions of six amino acids from Codon 444 to 449 (p. Pro444_Leu449del; ENST00000396625). The identified deletion was confirmed through Sanger sequencing. Further analyses and assessments were performed using disease databases such as ClinVar, ExAC and gnomAD (exome), and OMIM. The ClinVar database results reported that variant is pathogenic or likely pathogenic. The variant has not been reported in the 1000 genome database and has a minor allele frequency of 0.01% and < 0.01% in the ExAC and GnomAD (exome) databases. The results of OMIM phenotype found that homozygous or compound heterozygous variations in the *COL4A4* gene (OMIM∗120131) cause autosomal recessive Alport syndrome‐2 (OMIM#203780) in patients. The deletion in the *COL4A4* gene was classified as pathogenic, using PM2, PP3, and PM4 as several criteria from the ACMG guidelines.

**Table 2:** *COL4A4* gene variation by whole‐exome sequencing.

Gene transcript	*COL4A4* (NM_000092.5)
**Variant**	c.1323_1340del (p.Pro444_Leu449del)
**Location**	Exon 20
**Zygosity**	Homozygous
**Population frequency**	Absent from 1000 genome; ExAC ~0.01%; gnomAD < 0.01%
**Inheritance**	Autosomal recessive
**Disease (OMIM)**	Alport syndrome 2, autosomal recessive (OMIM#203780)
**ClinVar**	Reported as pathogenic/likely pathogenic
**ACMG classification**	Pathogenic
**ACMG evidence**	PM2 + PP3 + PM4

### 3.3. In Silico Assessment of the Identified Genetic Variation′s Impact on Protein Function

**Figure 1:**
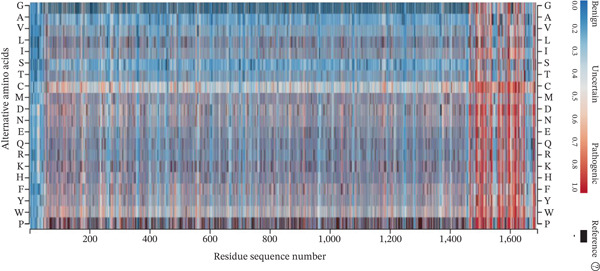
Heat map illustrating the pathogenicity scores for every possible single amino acid substitution within the COL4A4 protein. The *x*‐axis represents the order of the amino acid sequence of the COL4A4 protein, whereas the *y*‐axis shows the probability of basic 20 amino acid alternative substitutions (indicated by single‐letter codes), relative to the original residue. A color gradient from blue to red represents the predicted pathogenicity score, ranging from 0 (benign, blue) to 1 (pathogenic, red). The pathogenicity scores were predicted using the AlphaMissense model. Regions without color indicate positions where a missense mutation is not applicable or could not be scored.

The availability of in silico prediction tools such as the Mutation Taster tool, SIFT, PolyPhen2, FATHMM, and I‐Mutant2 has made it possible to annotate the impact of genetic variation(s) on the structure and function of a protein [11]. The genetic variation in the *COL4A4* gene was predicted to have an impact on protein structure and function when annotated by SIFT (Table 3). Similarly, the impact of this genetic variation was classified as probably damaging and damaging when predicted by PolyPhen2 and FATHMM. Protein stability was shown to decrease for the majority of proteins when analyzed by I‐Mutant2 (Table 3). AlphafoldDB Missense heat map evaluates the impact only for the substitution(s) introduced; therefore, the impact of deletion genetic variation in the *COL4A4* gene was not predicted by AlphafoldDB Missense (Figure 1). Based on these in silico predictions, it can be inferred that the reported genetic change may limit and/or damage the proper function of the protein.

**Table 3:** Evaluation of the impact of genetic change (c.1323_1340del) on COL4A4 protein native function.

In silico tool	Prediction
**SIFT**	Affects protein function
**PolyPhen2**	Probably damaging impact
**FATHMM**	Damaging
**I-Mutant2**	Stability decrease
**AlphafoldDB**	Not reported

### 3.4. PPIs via STRING Analysis

**Figure 2:**
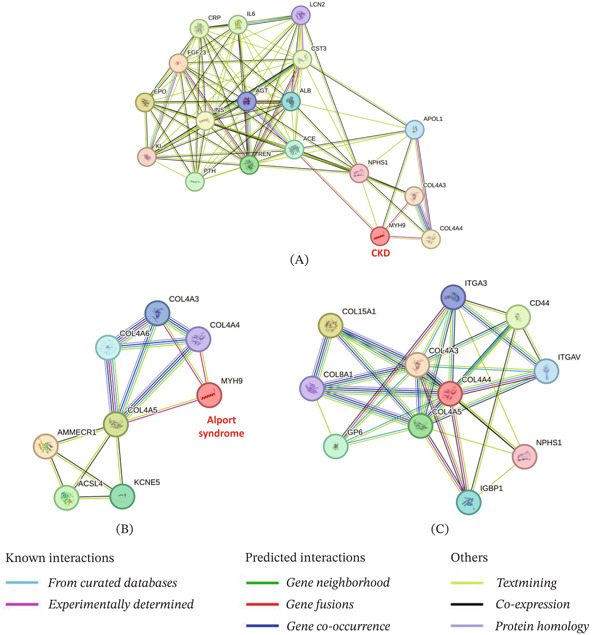
Annotated protein–protein interaction network. The disease–protein and protein–protein interactions network was created using the STRING database. (A) The disease–protein interaction network of chronic kidney disease (CKD), (B) The disease–protein interaction network of Alport syndrome (AS). (C) The protein–protein interaction of the COL4A4 network.

The protein interaction studies reveal potential interactions that are involved in executing specific functions [12]. Therefore, disease–protein interactions were performed to identify which proteins are associated with CKD and Alport syndrome using the STRING database. STRING network of CKD shows 18 nodes and 98 edges with the significant PPI enrichment *p* value of <1.0*e* − 16. The disease–protein interaction of CKD shows strong text mining and experimentally determined direct interactions with COL4A3 and COL4A4 proteins (Figure 2A). STRING network of Alport syndrome shows 8 nodes and 15 edges with the significant PPI enrichment *p* value of <1.0*e* − 16. The disease–protein interaction of Alport syndrome shows strong text mining and experimentally determined direct interactions with COL4A3, COL4A4, and COL4A5 proteins (Figure 2B). Further, we observe the STRING network of COL4A4 to identify which proteins are associated with this protein. COL4A4 STRING network shows 11 nodes and 36 edges with the significant PPI enrichment *p* value of 2.88*e* − 09. PPIs of COL4A4 shows interaction with COL4A3, COL15A1, CD44, COL4A5, GP6, ITGAV, COL8A1, and IGBP1 proteins (Figure 2C). Therefore, integrating genomic data with protein interaction networks provides a powerful approach to understanding the molecular mechanisms of protein interactions.

### 3.5. KEGG Analysis

KEGG analysis is a bioinformatics method for systematically linking genomic and molecular‐level information to higher order biological functions [14]. To understand the biological processes of CKD and Alport syndrome, KEGG pathways were studied. The pathways of CKD and Alport syndrome were studied using STRING KEGG pathway analysis and KEGG pathways (https://www.genome.jp/kegg/pathway.html).

KEGG pathway analysis shows that a total of 14 KEGG pathways were significantly associated with COL4A4 protein. These pathways are ECM–receptor interaction, protein digestion and absorption, small cell lung cancer, focal adhesion, human papillomavirus infection, PI3K‐Akt signaling pathway, pathways in cancer, AGE‐RAGE signaling pathway in diabetic complications, amoebiasis, relaxin signaling pathway, arrhythmogenic right ventricular cardiomyopathy, hematopoietic cell lineage, hypertrophic cardiomyopathy, and dilated cardiomyopathy.

**Figure 3:**
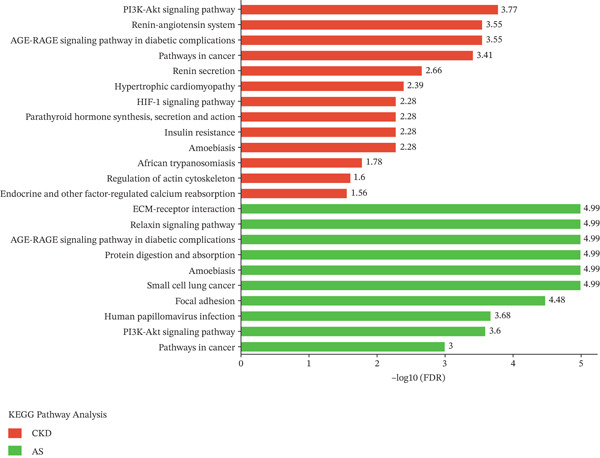
KEGG pathway analysis of human chronic kidney disease (CKD) and Alport syndrome (AS). The data of pathways enrichment were obtained from SRTING KEGG enrichment analysis, and bar graph was prepared on SR plot.

A total of 13 KEGG pathways were significantly associated with CKD. Among them, PI3K‐Akt signaling pathway, followed by renin‐angiotensin system, AGE‐RAGE signaling pathway in diabetic complications, pathways in cancer, renin secretion, hypertrophic cardiomyopathy, HIF‐1 signaling pathway, parathyroid hormone synthesis, secretion and action, insulin resistance, and amoebiasis were the most significant (Figure 3). A total of 10 KEGG pathways were significantly associated with Alport syndrome. Among them, ECM–receptor interaction, followed by relaxin signaling pathway, AGE‐RAGE signaling pathway in diabetic complications, protein digestion and absorption, amoebiasis, small cell lung cancer, focal adhesion, human papillomavirus infection, PI3K‐Akt signaling pathway, and pathways in cancer were the most significant (Figure 3). PI3K‐Akt signaling pathway, AGE‐RAGE signaling pathway in diabetic complications, pathways in cancer, and amoebiasis were consistent in both diseases, which shows the association among these diseases.

### 3.6. KEGG Pathway

**Figure 4:**
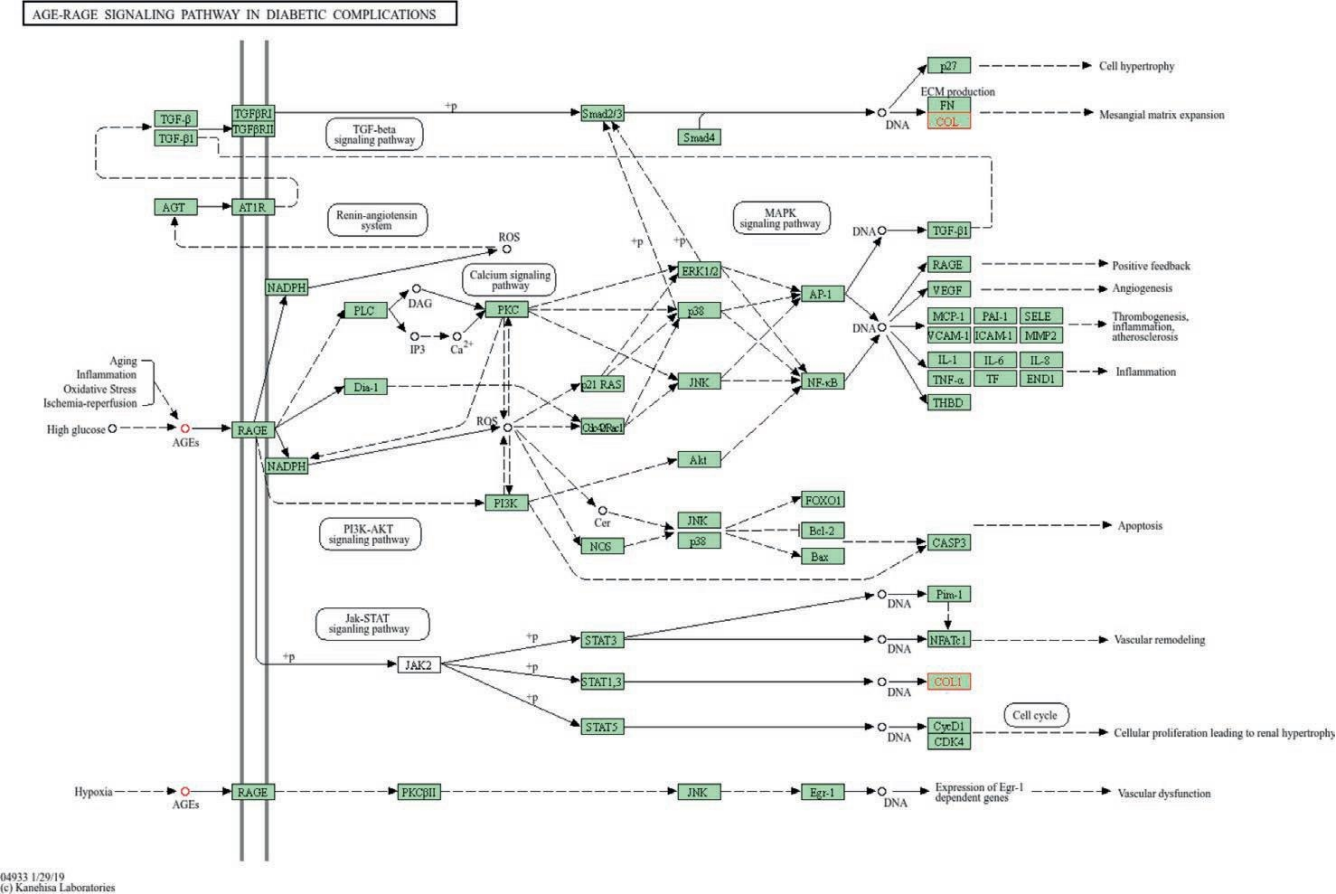
KEGG AGE‐RAGE signaling pathway: This figure illustrates the involvement of the *COL4A4* gene in the human AGE‐RAGE signaling pathway.

As AGE‐RAGE signaling pathway was significantly associated with CKD and Alport syndrome; therefore, this KEGG pathway was well studied. KEGG cytoskeleton of muscle cells pathway (https://www.kegg.jp/pathway/hsa04820) illustrates *COL4A4* links basement membrane integrity with cell survival, adhesion, and intercellular communication by integrating structural ECM components with intracellular signaling cascades. KEGG AGE‐RAGE signaling pathway illustrates COL family (including *COL4A4*) is a key component of the GBM and is susceptible to modification by advanced glycation end products (AGEs) under uremic conditions. The KEGG map further indicates that *COL4A4* indirectly interacts with downstream effectors of the receptor for advanced glycation end products (RAGE) (Figure 4). Activation of RAGE‐associated signaling pathways leads to the stimulation of molecular mediators involved in fibrosis, inflammation, and reactive oxygen species (ROS) generation, thereby contributing to renal injury and disease progression. In addition, JAK‐STAT–mediated and PI3K‐Akt–dependent pathways are induced via RAGE, which in turn participate in cell proliferation and apoptosis, respectively. Mutations in *COL4A4*, the structurally compromised ECM, which is also shown in the ECM–receptor interaction pathway, becomes highly susceptible to AGE accumulation and glycation‐induced damage, that enhances RAGE activation, amplifies downstream NF‐*κ*B and TGF‐*β* signaling, and accelerates podocyte injury and renal fibrosis, thereby linking diabetes‐associated AGE‐RAGE signaling with CKD progression in COL4A4‐related Alport syndrome.

## 4. Discussion

**Figure 5:**
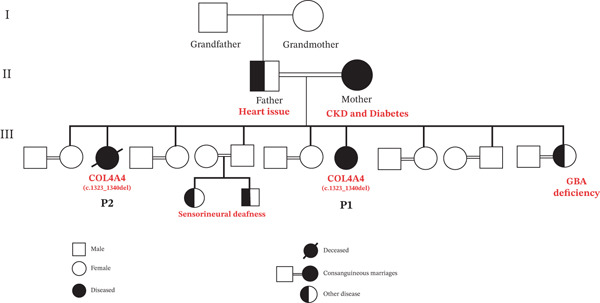
Family pedigree of the Alport syndrome patients showing autosomal recessive inheritance. Filled symbols represent affected or decreased individuals.

The participants in the current study were biological sisters who were diagnosed with Stage‐5 CKD. The girls and their family were found to be facing diabetes, heart problems, hypertension, and sensorineural deafness in addition to a kidney disorder. Diabetes, hypertension, heart disease, and obesity are reported to be the common risk factors for CKD [15]. The results of the ultrasound show that the kidneys were atrophied (shrunken) and brighter (fibrosis) due to CKD of Stage 5, which makes it difficult to determine the original cause of the kidney failure. Further, patients were not showing any signs of hearing loss and specific eye abnormalities. Therefore, genetic testing was performed to observe the actual cause of CKD. Genetic testing by WES revealed the mutation in the *COL4A4* gene in both patients. OMIM results reveal that the genetic variant in *COL4A4* is due to Alport syndrome 2, autosomal recessive (OMIM#203780). The deletion in the *COL4A4* gene could have caused the deletion of six amino acids, leading to shorter and dysfunctional collagen, which may, in turn, destabilize the entire collagen structure led to Alport syndrome. This deletion could have led to the weakening of the basement membrane in the kidneys, ears, and eyes, causing the primary symptoms of Alport syndrome. This may have led to renal failure or may also be accompanied by extrarenal alterations, such as sensorineural hearing loss [16]. Matthaiou et al. reported in a systematic review study that the GBM structure has been shown to be destabilized in Alport Syndrome due to *COL4A4* pathogenic variants (c.975G>T, c.2869G>C, c.1580delG, c.4494delG, and c.3648delG) that interfere with the production of *α*3*α*4*α*5 heterotrimers [17]. When we observe the pedigree and family history of patients, a niece and a nephew (siblings) exhibiting chromosomal abnormalities and diagnosed with sensorineural deafness which are the signs of Alport syndrome are existing in their family (Figure 5).

Identified variant (c.1323_1340del) was confirmed through several databases (Table 2). Differences between interaction databases such as ExAC, gnomAD, and 1000 Genomes arise because each resource integrates distinct types of evidence (experimental, predicted, or literature‐based), applies different curation strategies, and updates at different frequencies [11]. Consequently, the overlap between databases is partial, and not all interactions are uniformly reported. Further, databases are directly integrated into ACMG guidelines, where allele frequency information informs the criteria of pathogenic classification. Population frequency databases such as ExAC, gnomAD, and 1000 Genomes are highly accurate for determining allele frequencies, but they do not directly establish pathogenicity. In contrast, in silico prediction tools provide moderate accuracy in estimating the functional impact of variants. With advances in molecular biology, these in silico techniques are becoming less expensive and more accurate and may thus become easier for the general public to access [11]. In silico prediction tools like Mutation Taster, SIFT, PolyPhen2, FATHMM, and I‐Mutant2 are effective for assessing the effects of genetic variations on protein function and structure [11]. The mutation in the *COL4A4* gene (c.1323_1340del) in sisters was indicated to affect protein structure by SIFT and classified as damaging by PolyPhen2 and FATHMM. I‐Mutant2 analysis also showed decreased protein stability in the case of the *COL4A4* gene variant.

On follow‐up, P1 patient undergoes dialysis and kidney transplant, whereas P2 died because of kidney failure. Further, this study was carried out to investigate the association among CKD, Alport syndrome due to mutation in COL4A4 by disease–protein interaction, PPI and KEGG pathways (integrative molecular networks) that clearly shows the association among them by showing strong interaction with COL4A4 and lying in similar pathways. STRING analysis shows COL4A4 protein is strongly associated with both CKD and Alport syndrome diseases.

*COL4A* family (*COL4A1*, *COL4A2*, *COL4A3*, *COL4A4*, *COL4A5*, *COL4A6*) is a major component of the cell BM or ECM [18]. KEGG cytoskeleton of muscle cells pathway (https://www.kegg.jp/pathway/hsa04820) shows that proteins encoded by *COL4A* interact with muscle cell receptors by ECM–receptor interaction. *COL4A* is involved in ECM stability that impacts the integrity of muscle fibers, allowing efficient signal transduction from the ECM to the cytoskeleton [18]. Mutation in *COL4A* leads to BM weakening in the kidneys, ears, and eyes, causing the primary symptoms of Alport syndrome: progressive kidney disease, hearing loss, and ocular abnormalities [19].

*COL4A4* is part of a cooperative complex involving collagen, integrins, and adhesion molecules. By interfering with *COL4A4,* the integrity of the filtration barrier is diminished, and the molecular communication between ECM proteins and podocytes is compromised. *COL4A4* connections with CD44 and integrins suggest involvement in signaling and mechanosensing processes [20, 21]. Eliminating these interactions causes podocyte stress, which speeds up the development of glomerulosclerosis and Alport syndrome in CKD patients [21, 22]. STRING pathways analysis shows that the association of CKD and Alport syndrome in the AGE‐RAGE KEGG pathway. The role of *COL4A4* in the AGE‐RAGE KEGG pathway illustrates how Alport Syndrome and diabetic kidney disease may share mechanisms, particularly in the induction of fibrosis and oxidative stress. Pathogenic variants in *COL4A4* weaken GBM scaffolding, and in conjunction with AGE‐RAGE activation, the combined consequence is increased oxidative stress, inflammatory signaling, and overabundant ECM deposition [23]. The advancement of CKD and glomerular sclerosis is accelerated by this two‐hit injury. Clinically, it explains why individuals with COL4A4 variants typically show increasing fibrosis and renal degradation, which is comparable to what is seen in diabetic nephropathy. In Alport Syndrome, hereditary basement membrane defects and secondary profibrotic pathways combine to induce irreversible kidney injury [24], as demonstrated by the KEGG map of COL4A4 onto the AGE‐RAGE pathway. The patient observations corroborated the molecular analyses. Our patients with a homozygous deletion of COL4A4 (c.1323_1340del; p.Pro444_Leu449del) exhibited mesangial glomerulonephritis, hypertension, anemia, and rapid renal failure, with one sister eventually needing a transplant at the age of 20 years. The aggressive clinical progression mirrors the molecular findings: The disruption of COL4A4 destabilized the *α*3*α*4*α*5 trimer, hindered adhesion due to a loss of integrin signaling, and activated profibrotic AGE‐RAGE signaling, which collectively hastened the progression of CKD.

Variant interpretation in our study followed a multilayered approach: (1) detailed clinical evaluation, (2) whole‐exome sequencing for the identification of genetic variant, (3) Sanger sequencing for confirmation, (4) in silico pathogenicity prediction (SIFT, PolyPhen2, FATHMM, I‐Mutant2, and AlphaFold DB), and (5) network/pathway context analysis (STRING and KEGG). Interaction databases were used to provide biological context and hypothesis generation rather than as a standalone proof of pathogenicity. The p.(Pro444_Leu449del) deletion lies in the collagenous triple‐helical domain and is not located within an annotated residue‐level integrin‐binding motif. Nonetheless, the deletion of six residues in the triple helix is predicted to destabilize trimer assembly and may indirectly impair integrin or ECM interactions; residue‐level biochemical/structural testing (co‐IP, adhesion assays, and structural modeling) would be required to confirm altered binding.

It is summarized that genetic testing is the most reliable method to identify the unknown cause of CKD when kidney tissue is too scarred for imaging. As in our study, patients were in the Stage 5 of CKD, and the cause of kidney injury was not clear due to fibrosis. Therefore, genetic testing identify mutation in *COL4A4,* which is associated with Alport syndrome, even the symptoms of Alport syndrome were not clear in patients, but sensorineural deafness were observed in their family. Further, we find an association of *COL4A4* with CKD and Alport syndrome by STRING and KEGG pathways.

## 5. Conclusion

*COL4A4* plays a crucial role in preserving the stability of the GBM and ensuring podocyte adherence. Genetic pathogenic variants can disrupt this structural framework and trigger harmful signaling pathways, resulting in hematuria, proteinuria, and a swift advancement to end‐stage renal disease (ESRD) in individuals with Alport syndrome. Our clinical findings, especially from siblings with a *COL4A4* deletion, highlight how these molecular abnormalities lead to early‐onset hypertension, mesangial glomerulonephritis, and renal failure necessitating transplantation during adolescence. The combination of molecular network analyses with clinical findings shows that the pathology of Alport syndrome encompasses more than just structural abnormalities, incorporating oxidative stress, fibrosis, and immune system alterations. These revelations underline the importance of early genetic screening, family counseling, and the development of targeted therapies, including antifibrotic and antioxidant approaches to slow down disease progression and enhance patient outcomes.

## 6. Limitation

The patients were not suitable for a kidney biopsy due to CKD of Stage 5, as the kidneys were already shrunken in size. A biopsy of such tissue usually shows only nonspecific fibrosis, which provides little to no information. Therefore, biopsy and immunochemistry have not been done and these are the main limitations of our study.

## Funding

No funding was received for this manuscript.

## Conflicts of Interest

The authors declare no conflicts of interest.

## Data Availability

The data that supports the findings of this study are available from the corresponding authors upon reasonable request.
